# PPAR-*γ* Activation Alleviates Osteoarthritis through Both the Nrf2/NLRP3 and PGC-1*α*/*Δψ*_m_ Pathways by Inhibiting Pyroptosis

**DOI:** 10.1155/2023/2523536

**Published:** 2023-03-27

**Authors:** Zhencheng Feng, Qiuxiang Huang, Xingliang Zhang, Pengfei Xu, Siming Li, Dongli Ma, Qingqi Meng

**Affiliations:** ^1^Department of Orthopedics, Guangzhou Red Cross Hospital, Jinan University, Guangzhou 51022, China; ^2^Department of Respiratory Medicine, Guangzhou Twelfth People's Hospital, Guangzhou 510620, China; ^3^Department of Respiratory Medicine, Institute of Pediatrics, Shenzhen Children's Hospital, Shenzhen 518038, China; ^4^Department of Pediatrics, The Affiliated Hospital of Guangdong Medical University, Zhanjiang 524001, China; ^5^Department of General and Visceral Surgery, Surgery Center, Ulm University Hospital, Albert-Einstein-Allee 23, Ulm 89081, Germany; ^6^Guangzhou Institute of Traumatic Surgery, Guangzhou Red Cross Hospital, Jinan University, Guangzhou 510220, China

## Abstract

Osteoarthritis (OA) is a common degenerative joint disease with a gradually increasing morbidity in the aging and obese population. Emerging evidence has implicated pyroptosis in the etiology of OA and it may be recognized as a therapeutic target in OA. We have previously reported regarding another disease that peroxisome proliferator-activated receptor gamma (PPAR-*γ*) activation exerts an anti-inflammatory effect by suppressing the nucleotide-binding and oligomerization domain-like receptor containing protein (NLRP) 3 inflammasome. However, the relationship between PPAR-*γ* and NLRP3-mediated pyroptosis in OA cartilage and its underlying mechanisms is fully unclear. In this study, we found that the level of NLRP3-mediated pyroptosis in severe lateral femoral condyle cartilage wear in the knee of an OA patient was significantly higher than that in the mild lateral femoral condyle cartilage wear areas. Moreover, in lipopolysaccharide (LPS)/adenosine triphosphate (ATP)-induced primary chondrocytes and knee OA rat models, we demonstrated that activation of PPAR-*γ* by pioglitazone (Piog) attenuated LPS/ATP-induced chondrocyte pyroptosis and arthritis. These effects were partially counteracted by either blocking the nuclear factor erythroid-2-related factor (Nrf2)/NLRP3 or PGC1-*α*/*Δψ*_m_ signaling pathway. Simultaneous depression of these two signaling pathways can completely abrogate the protective effects of Piog on OA and chondrocytes. Taken together, Piog protects OA cartilage against pyroptosis-induced damage by simultaneously activating both the Nrf2/NLRP3 and PGC-1*α*/*Δψ*_m_ pathways, which enhances antioxidative and anti-inflammatory responses as well as mitochondrial biogenesis. Therefore, Piog may be a promising agent for human OA cartilage damage in future clinical treatments.

## 1. Introduction

Osteoarthritis (OA) is a chronic and degenerative joint disease without curative treatment. The prevalence of OA has a gradual ascent tendency for the increasingly aging population and obesity prevalence. The pathological features of OA mainly include cartilage destruction and synovial inflammation [1, 2]. Cartilage destruction during the development of OA always appears in joints, which mainly occurs in knee, hip, hand, and spine. Increasing evidence suggests that pro-inflammatory cytokines play a pivotal role in the degeneration of the articular cartilage matrix [3]. Interleukin (IL)-1*β* and IL-18 are mainly activated by caspase-1, which is central to pyroptosis [4]. Low-dose indomethacin and Hedgehog signal inhibitors could synergistically blunt the levels of caspase-1, IL-1*β*, and IL-18, which diminished cartilage damage in OA mice [5]. It appears a causal relationship exist between OA cartilage destruction and pyroptosis, but the exact mechanisms remain unclear.

Pyroptosis is a new type of programmed cell death. The activated caspase-1 as the core executor of pyroptosis is catalyzed by inflammasome [6]. The term “inflammasome” was first coined in 2002, and then four major inflammasomes, such as nucleotide-binding and oligomerization domain-like receptor containing protein (NLRP) 1, NLRP3, NLRC4, and AIM2, was characterized [7]. The NLRP3 inflammasome is a protein complex composed of NLRP3, an apoptosis-associated speck-like protein, and caspase-1, which is an attractive target for OA therapy [8]. Exogenous stimuli, such as lipopolysaccharide (LPS), and endogenous injury signals, such as uric acid and adenosine triphosphate (ATP) may induce reactive oxygen species (ROS) to activate the NLRP3 inflammasome and then trigger caspase-1-dependent canonical pyroptosis [9, 10]. The NLRP3 inflammasome can promote the expression of pro-inflammatory cytokines and degrading enzymes. IL-18 and matrix metalloproteinases, which are related to the pathogenesis of arthritis, can cause cartilage degeneration and synovial inflammation [8]. In experiments on fibroblast-like synovial cell and animal OA models and patients with knee OA, NLRP3 plays an important role in OA by regulating the inflammasome composition and the related downstream gene expression in the activation pathway of pyroptosis [11, 12]. Inhibiting NLRP3 can block pyroptosis, thus ameliorating chondrocyte injury and reducing OA occurrence. This indicates that anti-inflammatory therapy targeting the NLRP3 inflammasome might become a potential therapeutic target for OA treatment. Although there are many studies on NLRP3 in pyroptosis, they are still in the primary stage, especially regarding the mechanisms of OA. Therefore, it is necessary to further explore the relationship between NLRP3 and OA.

The imbalance in the redox state in the joint environment is the main cause of OA. Oxidative stress induced by ROS may be the predominant factor of NLRP3 inflammasome activation and downstream factor release in the development of OA [13, 14]. It has been reported that the increase in ROS caused by mitochondrial dysfunction is closely related to chondrocyte pyroptosis [15]. Mammalian cells have established a unique antioxidative defense system. Particularly, peroxisome proliferator-activated receptor gamma (PPAR-*γ*) and transcription factor nuclear factor erythroid-2-related factor (Nrf2) play a critical role in overcoming ROS-induced oxidative damage [16]. PPAR-*γ* is a key player in various biological processes, such as glycolipid metabolism, anti-inflammation, and cell proliferation and differentiation in multiple tissues [17]. A ligand of PPAR-*γ*, pioglitazone (Piog), is widely known as a therapeutic agent for treating type 2 diabetes [18]. Nrf2 triggered the endogenous defense mechanisms and mitochondria protective functions [19–21]. To eliminate cell damage caused by excessive ROS production, chondrocytes trigger endogenous defense to induce catalase and superoxide dismutase (SOD), which scavenge ROS through the Nrf2 pathway [22–24]. We have previously reported that PPAR-*γ* activation exerts an anti-inflammatory effect by suppressing the NLRP3 inflammasome in spinal cord-derived neurons [25]. However, the relationship between PPAR-*γ* and Nrf2 and the NLRP3 inflammasome in OA cartilage and its underlying mechanisms is not fully understood.

The functional interplay between PPAR-*γ* and its coactivator-1*α* (PGC-1*α*) is beneficial to antioxidative stress, anti-inflammation, and mitochondrial biogenesis [26, 27]. The anti-neuroinflammation of a synthesized non-thiazolidine-based glitazone, comparable with Piog, may be attributed to PPAR-*γ*-dependent PGC-1*α* signaling activation [28]. PGC-1*α* knockdown counteracted the effect of Piog on Mn-SOD levels and *Δψ*_m_ collapsed in hydrogen peroxide (H_2_O_2_)-treated HL-1 cells, which confirmed that Piog decreased diabetes-induced mitochondrial ROS through PPAR-*γ*, activating PGC-1*α* [29]. ROS production and mitochondrial damage were essential for NLRP3 inflammasome activation [30, 31], which was linked to cartilage degradation in OA [32]. Previous studies have clarified that ROS plays a role in inflammatory cytokine production in response to LPS [33, 34], which has also been shown to plays an important role in the activation of the NLRP3 inflammasome [35, 36]. However, the effects of the interaction between PPAR-*γ* and PGC-1*α* in OA pathogenesis are unclear.

In this study, we found that the level of NLRP3-mediated pyroptosis in the cartilage of severe OA patients was significantly higher than that in mild OA patients. In addition, in the LPS/ATP-induced OA rat model and primary chondrocyte experiments, we proved that PPAR-*γ* activation by Piog only partially attenuated LPS/ATP-induced pyroptosis of chondrocytes by blocking either the Nrf2/NLRP3 or PGC1-*α*/*Δψ*_m_ signaling pathway. Simultaneous depression of these two signaling pathways can completely abrogate the protective effects of Piog on OA, which demonstrates that the Nrf2/NLRP3 and PGC1-*α*/*Δψ*_m_ signaling pathways act together in PPAR-*γ* activation by attenuating pyroptosis in OA.

## 2. Materials and Methods

### 2.1. Reagents

Piog (PHR1632), LPS (L2880), GW9662 (M6191), ATP disodium salt hydrate (FLAAS), and dimethylsulphoxide (DMSO, D2650) were acquired from Sigma. MSU ((specific inhibitor against NLRP3; ab14430) was acquired from Abcam. 3-(4,5-Dimethylthiazol-2-yl)-5-(3-carboxymethoxyphenyl)-2-(4-sulfophenyl)-2H-tetrazolium (MTS) using CellTiter 96^®^ AQueous One Solution Cell Proliferation Assay kit was obtained from Promega (Madison, WI, USA) and Cell-Counting Kit-8 (CCK-8) assays were purchased from Dojindo (Kumamo, Japan). Mitochondrial membrane potential assay kit with JC-1 was obtained from Beyotime Biotechnology (C2006, Shanghai, China). Primary antibodies included: anti-NLRP3 (BA3677, BOSTER, Wuhan, China), anti-caspase-1 P10 (PA5-105049, Thermo Fisher Scientific, Waltham, MA, USA), anti-cleaved-GSDMD (GSDMD-N; ER1901-37, HUABIO, Hangzhou, China), anti-Nrf2 (AF7623, Beyotime Biotechnology), and anti-glyceraldehyde-3-phosphate dehydrogenase (GADPH; ARG10112, Arigobio, Shanghai, China). The peroxidase conjugated goat anti-mouse immunoglobulin G (IgG) (H + L) secondary antibody (AP124P) and goat anti-rabbit IgG-horseradish Peroxidase (HRP) secondary antibody (AP132P) were obtained from Millipore. Enzyme-linked immunosorbent assay (ELISA) kits were obtained from R&D systems (Minneapolis, MN, USA) for IL-1*β* and IL-18. Foetal bovine serum (FBS) and Dulbecco's modified Eagle's medium (DMEM)/Ham's F12 medium were purchased from Gibco (Grand Island, NY, USA). The PGC-1a inhibitor SR-18292 (SR) was obtained from MedChemExpress (HY-101491, South Brunswick Township, NJ, USA). DAB kit (chromogenic substrate for HRP) was obtained from Servicebio (G1212-200 T, Wuhan, China). Collagenase type II (C6885, Merck, Darmstadt, Germany). DMEM-F12 medium (113300032, Gibco/Life Technologies, Grand Island, NY, USA). FBS (10099141, Gibco/Life Technologies). One percent penicillin/streptomycin (15140-122, Gibco/Life Technologies). CellTiter 96^®^ AQueous One Solution Cell Proliferation Assay kit (Promega) for MTS assay. SuperSignal™ West Pico Pico PLUS (34579, Thermo Fisher Scientific) for detecting polyvinylidene fluoride (PVDF) membranes. ROS-GloTM H_2_O_2_ Assay kit (G8820, Promega). Nrf2 small interfering RNA (siRNA) (sense: GCUCAGAACUGUAGGAAAATT; antisense: UUUUCCUACAGUUCUGAGCTT) and its negative control (NC)-siRNA (sense: UUCUCCGAACGUGUCACGUTT; antisense: ACGUGACACGUUCGGAGAATT). Transfection reagent Lipofectamine 3000 (Invitrogen, Carlsbad, CA, USA; Lipofectamine™, L3000015).

### 2.2. Samples and Ethical Approval

Articular cartilage was obtained from patients who suffered from OA and received total knee arthroplasty (six women, age 64.33 ± 4.76 years). Patients with mild (*n* = 3) and severe lesions (*n* = 3) of knee lateral femoral condyle were enrolled at the Department of Orthopaedics at the Guangzhou Red Cross Hospital (Guangzhou, China). The study was approved by the Ethics Committee of the Guangzhou Red Cross Hospital (2017-001-01). All procedures involving animals were approved by the Laboratory Animal Centre of Jinan University (Guangzhou, China). Animal Care and Use Committee and conform to the Guide for Care and Use of Laboratory Animals.

### 2.3. Animals

Sprague–Dawley (SD) rats (8-week-old, 300–320 g, female) were purchased from Guangdong Laboratory Animal Center and housed in an environment (25°C, 70% humidity, 12 hours of light) with free access to food and water. Rats were randomly divided into the control, Piog, OA, and OA + Piog groups (*n* = 6 per group). In the OA group, a knee OA model was established using the Hulth method [37]. In brief, the rats were anesthetized via intraperitoneal injection of 0.3% pentobarbital sodium (10 ml/kg, Sigma). After opening the right knee joints, the articular cavity was exposed and then anterior cruciate ligaments, medial collateral ligaments, and medial menisci were cut off. The surgery was confirmed via drawer test. In the control group, rats underwent sham operation with joint cavity surgery, but no disturbance. In the Piog group, rats underwent sham operation with joint cavity surgery. After three weeks, rats were intraarticularly injected with 0.1 ml Piog (20 mM) twice a week for two weeks. In the OA + Piog group, knee OA model was established, and then rats were intraarticularly injected with 0.1 ml Piog (20 mM) twice a week for two weeks.

### 2.4. Magnetic Resonance Imaging Examination and Score of Cartilage Damage

The injury degree of knee OA was examined by magnetic resonance imaging (MRI; 1.5T, Avanto, I-Class, SIEMENS, Munich, Germany). The relative parameters for MRI scanning knee joints were set as TR/TE = 3000/30, slice thickness 3 mm, and base 320 × 320 matrix.

Cartilage signal and morphology were scored in the lateral femur centra of femorotibial joint using the fat-suppressed T2-weighted fast spin echo (FSE) images with an 8-point scale: 0 = normal thickness and signal; 1 = normal thickness and increased signal on T2-weighted images; 2.0 = partial thickness and focal defect <1 cm in greatest width; 2.5 = full thickness and focal defect <1 cm in greatest width; 3 = multiple areas of partial-thickness (Grade 2.0) defects intermixed with areas of normal thickness, or a Grade 2.0 defect wider than 1 cm but <75% of the region; 4 = diffuse (≥75% of the region) partial thickness loss; 5 = multiple areas of full thickness loss (grade 2.5) or a grade 2.5 lesion wider than 1 cm but <75% of the region; and 6 = diffuse (≥75% of the region) full thickness loss. Those MRI scores less than or equal to 2 points were selected as the mild group, and those scores greater than 2 point were selected as the severe group.

### 2.5. Primary Rat Chondrocyte Culture

The resting zone of the distal femoral growth plate of 2-day-old female SD rats were obtained for primary chondrocyte culture. Sequential digestion was performed by 0.25% trypsin/ethylene diamine tetraacetic acid (EDTA) (Invitrogen) for 1 hour at 37°C followed by 0.3% collagenase type II for 4 hours at 37°C. Then, cells were cultured in DMEM-F12 medium containing 10% FBS and 1% penicillin/streptomycin at 37°C and 5% CO_2_ till 80% confluence. The medium was changed every second day in CO_2_ incubator. Cells were resuspended in DMEM/F12 medium supplemented with 10% FBS and 1% penicillin/streptomycin and plated in monolayer at a density of 2 × 10^5^ cells/cm^2^.

### 2.6. Histopathology

Knee joint specimens were rinsed and fixed in 4% paraformaldehyde (Sigma) for 24 hours and then transferred to 10% EDTA. Next, the specimens were dehydrated then embedded in paraffin. Five micrometer sections were measured for NLRP3, caspase-1 P10, and GSDMD-N by immunohistochemistry. Quantification of staining optical intensities was performed with the NIH software Image-Pro Plus 6.0, as we previously described [25, 38].

The articular cartilage of rats was obtained for histomorphometric evaluations by using Safranin O/fast green stains (S–O staining). Depletion of proteoglycan and degeneration of cartilage were evaluated using the OA Research Society International (OARSI) system [39] (Table 1).

### 2.7. CCK-8 Assay

CCK-8 assay was used to evaluate the toxicity of Piog, LPS/ATP, and other reagents on chondrocytes. Briefly, chondrocytes were inoculated in a 96-well plates at a density of 4000/well, and cells were pretreated with Piog (10, 20, and 40 *μ*M) for 2 hours before stimulation with LPS/ATP for 24 hours. In other assays, GW9662 (PPAR*γ* antagonist), MSU, or SR-18292 (inhibitor against PGC-1*α*) was added to the medium as indicated. The cells were pretreated with a series of diluted Piog with or without LPS (1 *μ*g/ml)/ATP (5 mM) at final concentration. Experiments were divided into the following six groups: (1) control (treated with DMSO), (2) LPS/ATP group (LPS: 1 *μ*g/ml, ATP: 5 mM), (3) Piog + LPS/ATP group (Piog: 20 *μ*M, LPS: 1 *μ*g/ml, ATP: 5 mM), (4) Piog + GW + LPS/ATP group (Piog: 20 *μ*M, GW9662: 10 *μ*M, LPS: 1 *μ*g/ml, ATP: 5 mM), (5) Piog + MSU + LPS/ATP group (Piog: 20 *μ*M, MSU: 150 *μ*g/ml, LPS: 1 *μ*g/ml, ATP: 5 mM), and (6) Piog + SR + LPS/ATP group (Piog: 20 *μ*M, SR: 20 *μ*M, LPS: 1 *μ*g/ml, ATP: 5 mM). Cells were pre-treated with or without GW9662 for 6 hours, Piog for 2 hours, then LPS for 24 hours, and finally ATP for an additional 0.5 hour. In the Piog + MSU + LPS/ATP groups, MSU was pre-treated for 0.5 hour before Piog treatment. In the LPS/ATP + Piog + SR groups, SR was pre-treated for 2 hours before Piog treatment. The cells were incubated with 10 *μ*l of CCK-8 solution for an additional 4 hours at 37°C. The optical densities of the samples were measured at 450 nm on a spectrophotometer (Leica Microsystems, Wetzlar, Germany).

### 2.8. Cell Viability and Coefficient Assays

Rat chondrocytes were cultured till 80% confluence and then allocated in a 96-well plates at about 4 × 10^3^ cells/well. Piog dissolved in DMSO as a stock solution was diluted with phosphate buffered saline (PBS) for experiments, and the final concentration of vehicle in media was maintained at 0.1% (v/v). The cells were treated with a series of diluted Piog with final concentrations of 0, 5, 10, 20, 40, 80, and 160 *μ*M (equivalent volume of 0.1% DMSO in PBS as control) culture. The treatments of cells were set in three replicates and cultured at 37°C with 5% CO_2_ supplied in the incubator for 24 hours. The cell viability was measured by MTS assay. The MTS tetrazolium compound is bioreduced or transformed by metabolically active cells into a colored soluble formazan product in culture medium measurable by absorbance at 490 nm with a 96-well plates reader. These chondrocytes in 200 *μ*l were mixed with 40 *μ*l of MTS reagent for 4 hours incubation. The absorbance was measured using GloMax Multi + Detection System (Promega).

In coefficient assays, rat chondrocytes (4 × 10^3^ cells/well) were seeded in 96-well plates for 24 hours. The cells were pretreated with Piog (10, 20, and 40 *μ*mol/l) for 2 hours and primed with 1 *μ*g/ml LPS for 24 hours and subsequent stimulation with 5 mM ATP (LPS/ATP) for an additional 30 minutes. Then, the MTS assay was measured for cell viability.

### 2.9. Enzyme Linked Immunosorbent Assay

The productions of IL-1*β* and IL-18 were determined by the ELISA kit.

### 2.10. Western Blot Analysis

Western blot analysis was performed to examine the expression levels of indicated proteins in rat chondrocytes. Cells were lysed in radio-immunoprecipitation assay (RIPA) buffer containing protease inhibitors and centrifuged at 12,000× *g* for 10 minutes and the protein concentration was measured by a Bicinchoninic acid protein Assay (23227, Thermo Fisher Scientific, USA RIPA). Proteins were separated using 10% sodium dodecyl sulfate (SDS)/polyacrylamide gel electrophoresis and then transferred onto pPVDF membranes. After blocking with tris-buffered saline and tween-20 (TBST) buffer containing 5% bovine serum albumin (BSA) (v/v), the membranes were incubated with the primary antibodies overnight at 4°C, followed by the matched secondary antibody. PVDF membranes were detected using enhanced chemiluminescence and their signals were captured and analyzed using the Image Lab (Beta 1) Version 3.0.1 Changelist 40296 software. The expressional value of proteins was normalized to the intensity level of GADPH and proteins compared with the untreated cells (control) using the ImageJ software.

### 2.11. Detection of Mitochondrial Membrane Potential (*Δψ*_m_)

The *Δψ*_m_ maintains an electrochemical gradient across the mitochondrial membrane, which collapses in the process of cell death. The changes of *Δψ*_m_ were detected using the *Δψ*_m_ assay kit with JC-1 staining (Beyotime). The unique cationic dye, JC-1 (5,5,6,6-tetrachloro-1,1,3,3 tetraethylbenzimidazolylcarbocyanine iodide), is a membrane potential-sensitive probe that forms aggregates in high *Δψ*_m_ emitting a red fluorescent with a maximum at 590 nm. With collapsed *Δψ*_m_, monomers of JC-1 in a monomeric form emit a green fluorescent with a maximum at 530 nm. The intensity of the fluorescence is proportionally reflecting the *Δψ*_m_ level. The ratio between 530 and 590 nm emission manifests the fettle of *Δψ*_m_. Chondrocytes were allocated in a 6-well plates at about 2 × 10^5^ cells/well in DMEM-F12 medium containing 10% FBS and 1% penicillin/streptomycin. The pyroptosis of chondrocytes was induced by LPS/ATP and then treated with Piog, MSU, or SR as described above. After reaching drug action time, the treated chondrocytes were washed with PBS once, and 1 ml of JC-1 staining solution (50 *μ*l of C2006-1 was mixed with 8 ml of C2006-2 and 2 ml of C2006-3), then incubated at 37°C with 5% CO_2_ for 20 minutes. Cells were washed with 2 ml of 1× JC-1 solution twice before resuspended in 1 ml of DMEM-F12 medium for fluorescent detection. The fluorospectrophotometer (GloMax Multi Detection System (Promega)) was used to measure the signal (excitation at 490 nm and emission at 510–570 nm for monomers (green), excitation at 525 nm and emission at 580–640 nm for aggregates (red)) as instructed by the manufacturer. The ratio of red to green fluorescence in JC-1 stained cells was used for defining changes of *Δψ*_m_.

### 2.12. Measurement of ROS Generation

Experiments were divided into the following five groups: (1) control (treated with DMSO), (2) LPS/ATP group (LPS: 1 *μ*g/ml, ATP: 5 mM), (3) Piog + LPS/ATP group (Piog: 20 *μ*M, LPS: 1 *μ*g/ml, ATP: 5 mM), (4) Piog + GW + LPS/ATP group (Piog: 20 *μ*M, GW9662: 10 *μ*M, LPS: 1 *μ*g/ml, ATP: 5 mM), and (5) Piog + SR + LPS/ATP group (Piog: 20 *μ*M, SR: 20 *μ*M, LPS: 1 *μ*g/ml, ATP: 5 mM). Cells were pre-treated with or without GW9662 for 6 hours, Piog for 2 hours, then LPS for 24 hours, and finally ATP for an additional 0.5 hour. In the LPS/ATP + Piog + SR groups, SR was pre-treated for 2 hour before Piog treatment. The production of intracellular ROS was assessed using the ROS-GloTM Assay kit, as we previously described [24].

### 2.13. Nrf2 siRNA Transcription

Nrf2 siRNA and NC-siRNA (Supplementary Data) were synthesized by Guangzhou Hanyi Biological Technology Co., Ltd. A total of 2 × 10^5^ rat chondrocytes were added to each well of a 6-well culture plate and incubated overnight. Transfection of Nrf2-siRNA and NC-siRNA into chondrocytes with Lipofectamine 3000 according to the instructions provided by Invitrogen. After further treatment, cells were harvested for biochemical detections.

### 2.14. Statistical Analysis

Each experiment was performed in triplicate and expressed as mean ± SD. Statistical analysis was conducted using the SPSS Statistics 20 software. Significant differences between groups were determined using a Student's *t*-test and an unpaired one-way ANOVA test. Statistical significance was set at *P* < 0.05.

## 3. Results

### 3.1. The Level of NLRP3-Mediated Pyroptosis in the Cartilage of OA Patients Correlated Positively with OA Severity

**Figure 1:**
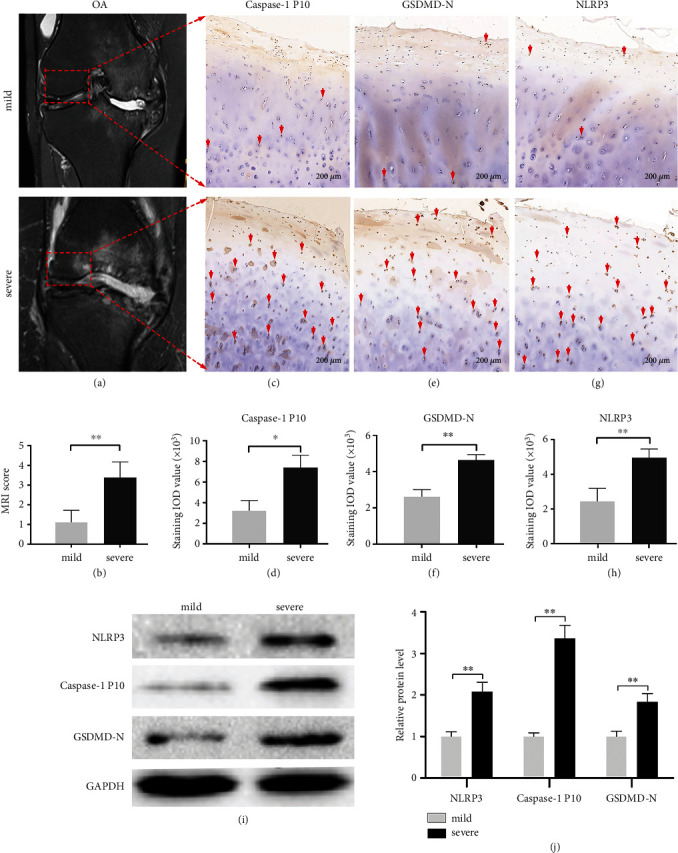
The upregulated levels of NLRP3, caspase-1 P10, and GSDMD-N in the cartilage of the lateral femoral condyle of the knee of a patient with severe OA. (a and b) MRI shows the lateral femoral condyle of the human knee, which has mild and severe lesions. MRI scoring was used to assess injury severity. (c–h) The levels of caspase-1 P10, GSDMD-N, and NLRP3 in mild and severe knee osteoarthritis are shown using immunohistochemical staining (bar: 200 *μ*m). Quantification of the immunohistochemical staining is located below. (i and j) Representative immunoblots of NLRP3, caspase-1 P10, and GSDMD-N in the mild and severe lesion groups. GAPDH was used as an internal reference. Densitometric analysis of the immunoblots is located on the right. The data represent three independent experiments. ∗*P* < 0.05 and ∗∗*P* < 0.01.

To determine whether pyroptosis is related to OA severity, the lateral femoral condyle of the human knee with mild or severe lesions (Figures 1(a) and 1(b)) was subjected to immunohistochemistry to detect pyroptosis. Activated caspase-1 is the core pyroptosis executor for producing mature GSDMD-N as well as IL-1*β* and IL-18 [6, 12]. Therefore, we first detected activated caspase-1 P10 and GSDMD-N levels, which were significantly higher in severe lesions than those in the mild lesions (Figures 1(c), 1(d), 1(e), and 1(f)). Moreover, the key signature of the classical activation pathway of pyroptosis is the formation of an inflammasome. NLRP3 is the most common component of inflammasome [8, 12]. As expected, we found that the NLRP3 level in the severe lesions was higher than that in the mild lesions (Figures 1(g) and 1(h)). Similarly, the western blotting analysis showed that the levels of these three proteins in the severe lesions were higher than those in the mild lesions (Figures 1(i) and 1(j); Table S1). These results indicate that NLRP3-mediated pyroptosis may be involved in the pathogenesis of OA.

### 3.2. Upregulated NLRP3 and Degenerating Symptoms in a Knee OA Model of Rats Can Be Reversed Through PPAR-*γ* Activation

**Figure 2:**
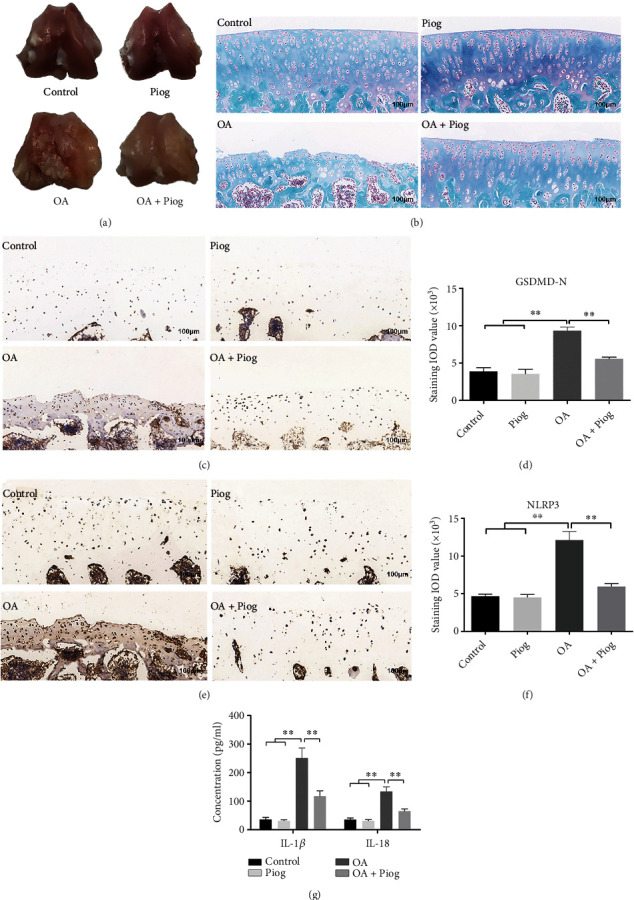
Piog alleviates osteoarthritis and reduces NLRP3-mediated pyroptosis in rat experiments. (a) Gross observation of the control, OA, Piog, and OA + Piog groups. (b) The rat femur coronals were sectioned and stained using S-O staining. The cartilage area is stained red and the subchondral bone blue. The sections of the control, OA, Piog, and OA + Piog groups were observed under the microscope (bar: 100 *μ*m). (c–f) Immunohistochemical staining was performed to detect the levels of GSDMD-N and NLRP3 in the lateral femoral condyle of the rat knee (bar: 100 *μ*m). The immunohistochemical staining results were quantified. (g) ELISA was used to measure the concentrations of IL-1*β* and IL-18 in the cartilage. The data represent three independent experiments and are expressed as mean ± SD. ∗∗*P* < 0.01.

After establishing a knee OA model of rats, we observed the pathological changes between the control and the OA groups. The articular surface in the control group was smooth. However, in the OA group, the articular surface of the femoral condyle became rough and pale with multiple osteophyte hyperplasia, local cartilage defects, and fissures (Figure 2(a)). The coronal tissue sections of the femoral condyle were stained using S-O staining for microscopic analysis. In the control group, the cartilage displayed a smooth surface and clear cell layers with ordered chondrocytes stained red, whereas the subchondral bone was stained green with normal morphology. In the OA group, the cartilage surface showed some defects; the chondrocytes were disordered, the sliding-band cells were absent, and the subchondral bone retained normal morphology in blue (Figure 2(b)). The OARSI score system was used to evaluate the depletion of proteoglycan and degeneration of cartilage (Table 1), showing a significant difference between the OA and control groups (Table 2). Next, the lateral femoral condyles in the control and OA groups were subjected to immunohistochemistry. We found that the levels of GSDMD-N (Figures 2(c) and 2(d)) and its upstream NLRP3 (Figures 2(e) and 2(f)) increased significantly in the OA group compared with the control group. As expected, the ELISA results showed that IL-1*β* and IL-18 levels in the cartilage increased significantly in the OA group (Figure 2(g)).

PPAR-*γ* plays an important role in alleviating inflammatory responses [17]. We previously reported that PPAR-*γ* activation suppressed the NLRP3 inflammasome in spinal cord-derived neurons [25]. Here, we explored whether PPAR-*γ* activation by Piog could inhibit NLRP3-mediated pyroptosis in the OA cartilage. The cartilage articular surface was smooth in the Piog group, similar to that in the control group. The articular surfaces of the femoral condyle became smoother, and the local cartilage surfaces were less pale in the OA + Piog group compared with the OA group (Figure 2(a)). Compared with the OA group, the pathological characteristics of the OA + Piog group improved significantly similar to the smoother cartilage surface. The chondrocytes were clearly and orderly arranged in red and the subchondral bone returned to normal morphology in green. The sliding-band cells were effectively rescued, but their density decreased compared with the control group (Figure 2(b)). The OARSI score in the OA + Piog group decreased compared with that in the OA group (Table 2). The immunohistochemistry results showed that Piog can inhibit the levels of GSDMD-N (Figures 2(c) and 2(d)) and NLRP3 (Figures 2(e) and 2(f)) in the OA cartilage. Compared with the OA group, IL-1*β* and IL-18 levels in the cartilage of the OA + Piog group increased significantly (Figure 2(g)).

### 3.3. PPAR-*γ* Activation Inhibits LPS/ATP-Induced Pyroptosis in OA Chondrocytes

**Figure 3:**
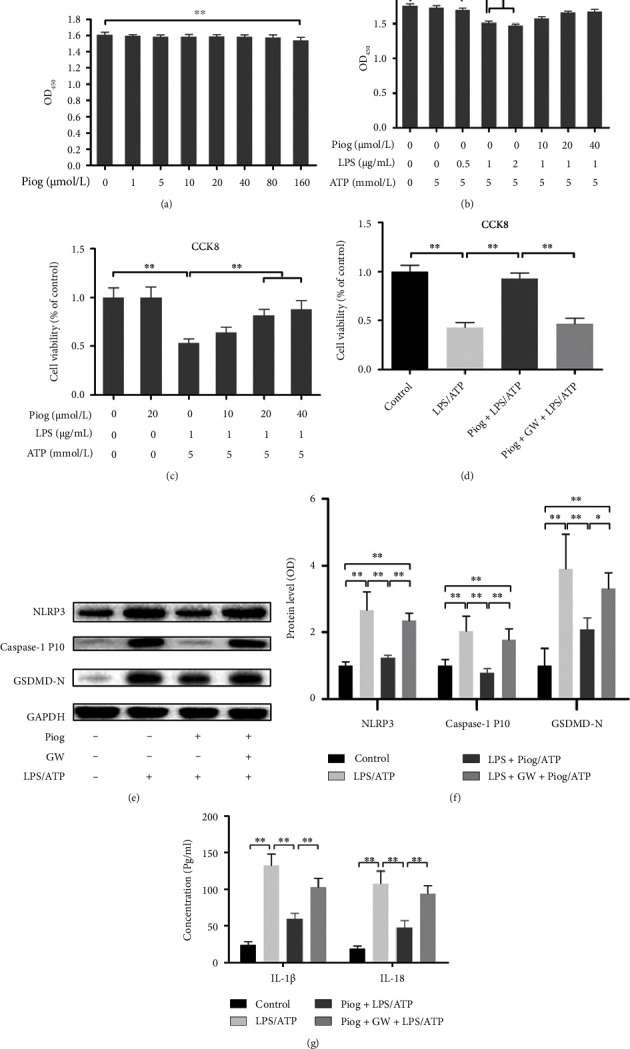
Piog attenuates LPS/ATP-induced pyroptosis of chondrocytes. (a) MTS assays were performed for cell toxicity after Piog treatment for 24 hours. (b and c) MTS and CCK8 assays were performed for rat chondrocyte viability. Piog pretreatment for 2 hours before LPS treatment for 24 hours and subsequent stimulation with ATP for 30 minutes. (d) CCK8 assays were performed for the effect of Piog on LPS/ATP-treated chondrocyte viability after GW antagonizing PPAR-*γ* using 20 *μ*M Piog, 1 *μ*M LPS, 5 mM ATP, and 10 *μ*M GW. (e and f) Western blot and densitometric analysis of the immunoblots for NLRP3, caspase-1 P10, and GSDMD-N in the control, LPS/ATP, Piog + LPS/ATP, and GW + Piog + LPS/ATP groups. GAPDH was used as an internal reference. (g) ELISA was performed for IL-1*β* and IL-18 concentrations in the control, LPS/ATP, Piog + LPS/ATP, and Piog + GW + LPS/ATP groups. The data represent three independent experiments and are expressed as mean ± SD. ∗*P* < 0.05 and ∗∗*P* < 0.01.

To further verify whether PPAR-*γ* activation can inhibit inflammasome NLRP3-mediated pyroptosis in the knee cartilage of OA, we used Piog (Figure S1) to activate PPAR-*γ* and then investigated the NLRP3-mediated pyroptosis in primary cultured rat chondrocytes. First, the MTS assay results showed the negligible toxicity of Piog (1–80 *μ*M) except 160 *μ*M on chondrocyte viability (Figure 3(a)). In addition, LPS stimulation of articular cartilage can simulate an inflammatory environment similar to OA [40]; therefore, cell viability was measured using MTS and CCK8 assays to screen the optimal concentrations of LPS/ATP and Piog in the pyroptosis model of rat chondrocytes. The MTS assay results showed that 0, 0.5, and 1 *μ*g/ml LPS exhibited a dose-dependent inhibition on cell viability, whereas 2 *μ*g/ml LPS exhibit less potent inhibition. Moreover, pretreatment with 10, 20, or 40 *μ*M Piog 2 hours before the administration of LPS/ATP reversed the inhibited chondrocyte viability in a dose-dependent manner (Figure 3(b)), which accorded with the results from the CCK8 assay (Figure 3(c)). A noncytotoxic concentration (20 *μ*M) of Piog was used for the subsequent experiments. Finally, after GW9662 inhibited PPAR-*γ*, Piog could not relieve the cell viability suppressed by LPS/ATP (Figure 3(d)).

Next, immunoblotting experiments were used to detect the inhibitory effect of PPAR-*γ* activation on LPS/ATP-induced chondrocyte pyroptosis. Compared with the control group, the levels of NLRP3, caspase-1 P10, and GSDMD-N increased in the LPS/ATP group. Piog reversed the above effects induced by LPS/ATP. After treatment with the PPAR-*γ* antagonist GW, the levels of NLRP3, caspase-1 P10, and GSDMD-N increased again (Figures 3(d), 3(e), and 3(f)). In the ELISA experiments, the levels of IL-1*β* and IL-18 in the Piog + LPS/ATP group were lower than those in the LPS/ATP group. After treatment with GW, the suppressed levels of IL-1*β* and IL-18 in the Piog + LPS/ATP group were massively restored (Figure 3(g)).

### 3.4. PPAR-*γ* Activation Partially Attenuates LPS/ATP-Induced Chondrocyte Pyroptosis Through the Nrf2/NLRP3 Pathway

**Figure 4:**
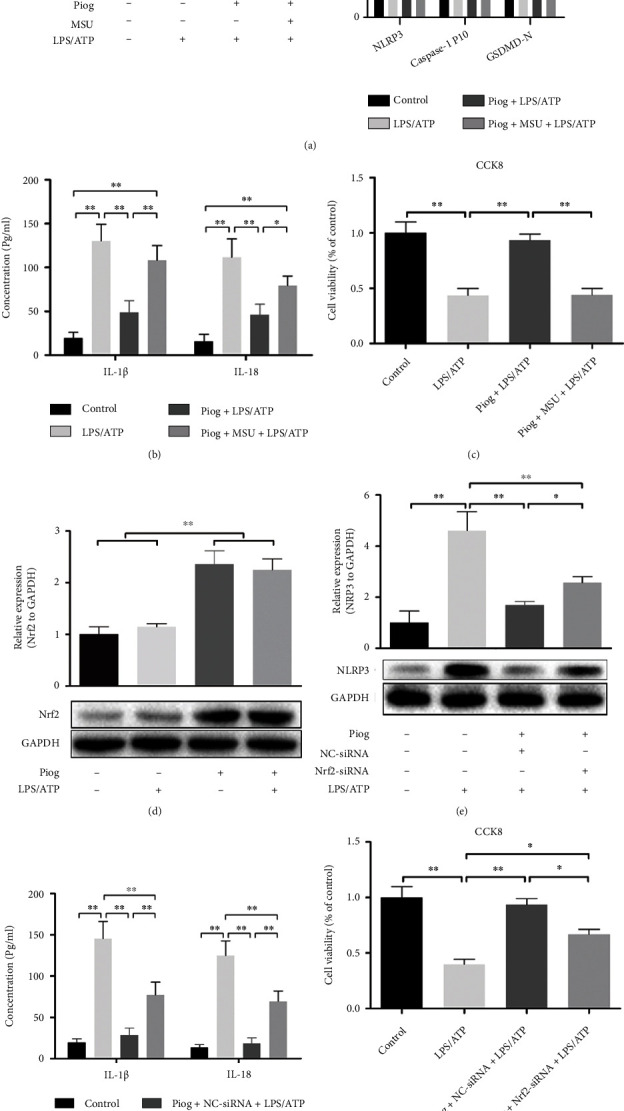
Piog partially attenuates LPS/ATP-induced chondrocyte pyroptosis related to the NLRP3 inflammasome through Nrf2 signaling. (a and b) Western blot analysis for NLRP3, caspase-1 P10, and GSDMD-N in the control, LPS/ATP, Piog + LPS/ATP, and Piog + MSU + LPS/ATP groups. (b) ELISA was performed for IL-1*β* and IL-18 concentrations. (c) CCK8 assays were performed to determine the effect of Piog on LPS/ATP-treated chondrocyte viability after MSU activated NLRP3. (d) Western blot analysis for Nrf2 in the control, LPS/ATP, Piog, and Piog + LPS/ATP groups. (e) Western blot analysis was performed to detect NLRP3 level after Nrf2 knockdown by siRNA; (f) ELISA was performed to detect IL-1*β* and IL-18 concentrations. (g) CCK8 assays were performed to determine the effect of Piog on LPS/ATP-treated chondrocyte viability after Nrf2 knockdown by siRNA. GAPDH was used as an internal reference for western blotting. Densitometric analysis of the immunoblots is located on the right or below. The data represent three independent experiments and are expressed as mean ± SD. ∗*P* < 0.05 and ∗∗*P* < 0.01.

To understand whether chondrocyte pyroptosis inhibited by PPAR-*γ* activation was mediated by NLRP3, chondrocytes were preincubated with the NLRP3 agonist MSU before Piog + LPS/ATP treatment. The levels of NLRP3, caspase-1 P10, and GSDMD-N were increased significantly after MSU stimulated NLRP3 (Figure 4(a)). The IL-1*β* and IL-18 levels in the chondrocytes were effectively rescued by MSU, which reversed the inhibitory effect of Piog on IL-1*β* and IL-18 levels (Figure 4(b)). Cell viability assays showed that the upregulation of NLRP3 levels by MSU also relieved the inhibitory effect of Piog on LPS/ATP-induced chondrocyte pyroptosis (Figure 4(c)).

The Nrf2 signaling pathway might be the key pathway to alleviate the NLRP3 inflammasome [14, 41]. Several studies have documented the existence of an interplay between the Nrf2 and PPAR-*γ* pathways that potentiates the levels of each other [16, 42, 43]. To determine whether the PPAR-*γ* activation-triggered attenuation of NLRP3-mediated pyroptosis correlates with Nrf2 activation, we examined the level of Nrf2 in response to Piog, LPS/ATP, or a combined modality. Western blotting experiments showed that LPS/ATP stimulation alone did not change the levels of Nrf2, whereas Piog increased Nrf2 levels regardless of the level of administration of LPS/ATP (Figure 4(d)). Knocking down Nrf2 with a specific siRNA partially reversed the inhibitory effect of Piog on NLRP3 levels in the LPS/ATP-induced chondrocyte pyroptosis (Figure 4(e)). There was a significant difference between the LPS/ATP and Piog + Nrf2-siRNA + LPS/ATP groups, which confirmed that Piog partially inhibited the NLRP3 inflammasome through the Nrf2 pathway (Figure 4(e)). Moreover, The ELISA experiments showed that the half levels of IL-1*β* and IL-18 inhibited by Piog were effectively rescued compared with those of the LPS/ATP group (Figure 4(f)), which was consistent with the trend of CCK8 assay results (Figure 4(g)).

### 3.5. PPAR-*γ* Activation Partially Attenuates LPS/ATP-Induced Pyroptosis of Chondrocytes Through the PGC1-*α*/*Δψ*_m_ Signaling Pathway

**Figure 5:**
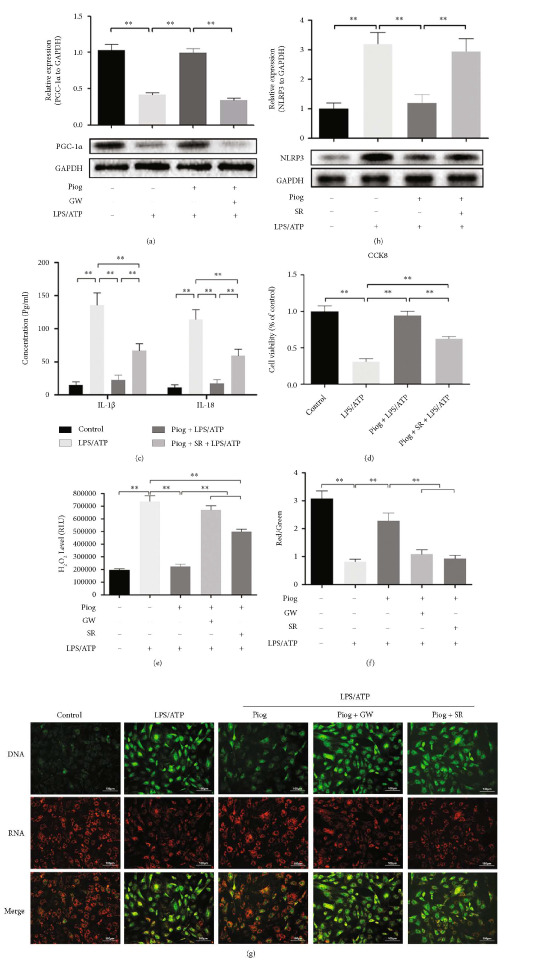
Piog partially attenuates LPS/ATP-induced pyroptosis of chondrocytes through the PGC1-*α*/*Δψ*_m_ signaling pathway. (a) PPAR-*γ* activation by Piog rescued PGC1-*α* expression in the presence of LPS/ATP. Western blot analysis for PGC1-*α* in the control, LPS/ATP, Piog + LPS/ATP, and Piog + GW + LPS/ATP groups. (b) Cells were pretreated with Piog in the presence or absence of SR and then stimulated with LPS/ATP. The level of NLRP3 was detected using western blotting. (c) ELISA was performed to measure IL-1*β* and IL-18 levels in the control, LPS/ATP, Piog + LPS/ATP, and Piog + SR + LPS/ATP groups. (d) CCK8 assays were performed to determine the chondrocyte viability in the control, LPS/ATP, Piog + LPS/ATP, and Piog + SR + LPS/ATP groups. (e) The intracellular ROS content was detected in the same five groups using *Δψ*_m_ assays. (f and g) The ratio of red fluorescent emission of cells with intact *Δψ*_m_ against green fluorescent emission of cells with impaired *Δψ*_m_ was plotted to represent the changes in *Δψ*_m_. Fluorescent spectrophotometry was used to detect the *Δψ*_m_ of JC-1-stained cells in the control, LPS/ATP, Piog + LPS/ATP, GW + Piog + LPS/ATP, and SR + Piog + LPS/ATP groups (bar: 100 *μ*m). GAPDH was used as an internal reference for western blotting. The characteristic immunoblot pictures are located under their densitometric analysis. The data represent three independent experiments and are expressed as mean ± SD. ∗∗*P* < 0.01.

Reports that PPAR-*γ* activation in response to Piog increased the PGC-1*α* level have been documented in other diseases [26, 27]. We wanted to determine whether PPAR-*γ* activation suppresses NLRP3 inflammasome-mediated pyroptosis of chondrocytes through the PGC1-*α* pathway. The western blot analysis showed that Piog rescued the LPS/ATP-induced inhibition of PGC1-*α* level, which was counteracted by treatment with the PPAR-*γ* antagonist GW (Figure 5(a)). After pretreatment with the PGC-1*α* antagonist SR, the levels of NLRP3 in the Piog + LPS/ATP-induced chondrocytes were partially restored (Figure 5(b)). Moreover, ELISA results showed that the inhibited levels of IL-1*β* and IL-18 in the presence of Piog + LPS/ATP were released by half in response to SR (Figure 5(d)). The CCK8 assays showed that SR could significantly compromise the cell viability protected by Piog. There were significant differences in NLRP3 levels (Figure 5(b)), IL-1*β* and IL-18 concentrations (Figure 5(c)), and cell viability (Figure 5(d)) between the SR + Piog + LPS/ATP and Piog + LPS/ATP groups, which indicate that PGC-1*α* partially, but not totally, mediated the antipyroptosis effect of Piog.

PGC1-*α* as a master regulator of mitochondrial biogenesis and function is used to ROS and stabilize *Δψ*_m_. Therefore, we detected ROS and *Δψ*_m_ to assess the effect of the SR antagonizing PGC1-*α* on mitochondria in the presence of Piog + LPS/ATP. First, the effect of Piog on intracellular ROS levels in cultured rat chondrocytes through the PGC-1*α* pathway was measured. LPS/ATP significantly increased the intracellular ROS content in chondrocytes, which was strongly counteracted by the administration of Piog. After pretreatment with the PPAR-*γ* antagonist or PGC-1*α* antagonist SR, the intracellular ROS content was restored compared with that in the Piog + LPS/ATP group. There was a significant difference in ROS content between the Piog + GW + LPS/ATP and Piog + SR + LPS/ATP groups, which indicate that the PGC-1*α* pathway only partly accounts for the inhibitory effect of Piog on ROS content caused by LPS/ATP (Figure 5(e)). In addition, we detected *Δψ*_m_ to assess the effect of the SR antagonizing PGC1-*α* on mitochondria in the presence of Piog + LPS/ATP. Fluorospectrophotometry analysis of JC-1-stained cells showed a high *Δψ*_m_ with strong intensity of red fluorescence in the control group and a low *Δψ*_m_ with strong intensity of green fluorescence in the LPS/ATP group. The red fluorescence intensity increased, but the green fluorescence decreased in the Piog + LPS/ATP group. The opposite fluorescence pattern was produced through pretreatment with the PPAR*γ* antagonist GW. After pretreatment with the PGC-1*α* antagonist SR, the fluorescence pattern was comparable with that in the Piog + GW + LPS/ATP group (Figures 5(f) and 5(g)). The variation of red/green fluorescence intensity, which indicates *Δψ*_m_, demonstrates that Piog played a critical role in stabilizing *Δψ*_m_ through the PGC-1*α* pathway. Taken holistically, Piog exerts its antipyroptosis effect partly through the PGC1-*α*/*Δψ*_m_ signaling pathway.

### 3.6. Nrf2 And PGC-1*α* Act Together in PPAR-*γ* Activation to Attenuate Pyroptosis of OA Chondrocytes

**Figure 6:**
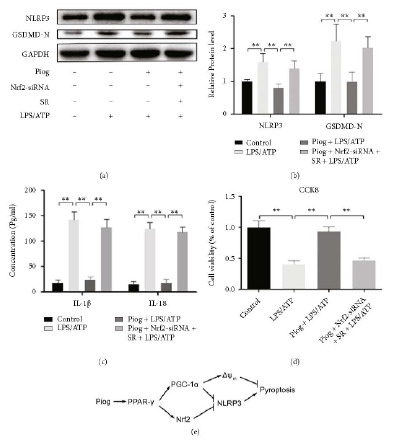
Involvement of Nrf2 and PGC-1*α* in the effect of Piog on pyroptosis in the OA chondrocytes. (a and b) Western blotting experiments and densitometric analysis. SR was used as a PGC-1*α* inhibitor. Nrf2-siRNA was used for knocking down the level of Nrf2. GAPDH was used as an internal reference. (c) ELISA was used to measure IL-1*β* and IL-18 concentrations. (d) CCK8 assays were performed to measure chondrocyte viability. ∗∗*P* < 0.01, *n* = 5. (e) The action mode of PPAR-*γ* under Piog stimulation to protect from pyroptosis-mediated OA cartilage damage.

After knocking down Nrf2 with siRNA and antagonizing PGC-1*α* with SR, the levels of NLRP3 and GSDMD-N in the chondrocytes were dramatically restored, and the inhibitory effect of Piog on LPS/ATP-induced pyroptosis was almost relieved (Figures 6(a) and 6(b)). Moreover, we assessed the levels of IL-1*β* and IL-18 using ELISA. We found that both Nrf2 and PGC-1*α* inhibition blocked the Piog-mediated antipyroptosis effects (Figure 6(c)), which was consistent with the results of the CCK8 assays (Figure 6(d)). These results suggest that PPAR-*γ* activation inhibited rat NLRP3-mediated pyroptosis in the OA chondrocytes through the combined action of the Nrf2 and PGC-1*α* signaling pathways (Figure 6(e)).

## 4. Discussion

OA, which is mainly characterized by progressive joint sclerosis and cartilage degeneration, is the most common chronic joint disease. In recent years, it has been found that pyroptosis is closely related to multiple local lesions of OA tissues, mainly including synoviocytes and chondrocytes. In the synovium of the rat anterior cruciate ligament transected knee OA model and in the LPS/ATP-induced fibroblast-like synoviocytes, the NLRP1/NLRP3 inflammasome-mediated pyroptosis increased [44]. Human fibroblast-like synoviocytes from knee OA patients were stimulated with LPS/ATP to induce pyroptosis, which was attenuated by NLRP1 and NLRP3 siRNAs [11]. In the monosodium iodoacetate (MIA)-induced knee OA of rats and the rat fibroblast-like synoviocytes, the upregulated HIF-1*α* may exacerbate synovial fibrosis through pyroptosis, and suppress the upregulation of HIF-1*α* could remit synovial fibrosis [45]. Moreover, in the cartilage of the OA model of rats induced by MIA and the chondrocytes induced by LPS, icariin could inhibit NLRP3-mediated pyroptosis [12]. In the surgical destabilization of the medial meniscus-induced OA model of mice and the primary mouse chondrocyte culture stimulated by tumor necrosis factor (TNF)-*α*, the pharmacological inhibition of NLRP3-mediated pyroptosis ameliorated inflammation in the chondrocytes and OA development [46]. As far as we know, we for the first time found that the level of NLRP3-mediated pyroptosis in the cartilage of the severe OA patients was more active than that in the mild patients, which is in agreement with the roles of NLRP3-mediated pyroptosis in the primary rat chondrocytes.

PPAR-*γ* and Nrf2 have been shown to play key roles in controlling inflammation and oxidative insults. Several lines of evidence have strongly indicated the activation of the Nrf2 pathway by PPAR-*γ*. PPAR-*γ* activation by the PPAR-*γ* agonist chrysin could promote Nrf2 levels and diminish oxidative stress, which was crucial for cardioprotection in an ischemia–reperfusion-induced myocardial infarction [47]. Rosiglitazone increased the level of Nrf2 and then eliminated high glucose-induced excessive ROS in a PPAR-*γ*-dependent manner [48]. Bioinformatics analysis identified a putative PPAR response element in the Nrf2 promoter [49]. The hyperoxia-induced upregulation of Nrf2 was reduced by silencing PPAR-*γ* in mice [42]. Nrf2 level was blocked by the knockdown of PPAR-*γ* in mouse liver by fasting [49]. Several studies have supported the existence of the opposite pattern of regulation, which is PPAR-*γ* induction of the Nrf2 pathway [16]. Hyperoxia-induced upregulation of PPAR-*γ* was markedly greater in wild-type mice than in Nrf2^−/−^ mice [50]. Nrf2 binds to the antioxidant response elements of the PPAR-*γ* promoter for induction of PPAR-*γ* in hyperoxia-induced acute lung injury [51]. Nrf2-knockdown attenuates PPAR-*γ* and Mn-SOD level during adipogenesis in 3T3-L1 cells [52]. These studies indicate that Nrf2 and PPAR-*γ* might reciprocally potentiate the expression of each other, presumably under basal and pathological conditions. Nevertheless, an opposite expression variation between Nrf2 and PPAR-*γ* has been discovered. Vinpocetine as a nootropic drug attenuated Nrf2 and increased PPAR-*γ* level in fluoxetine-induced liver damage of rats [53]. PPAR-*γ* can regulate the endogenous catalase [54]. We previously reported that catalase may protect chondrocytes from apoptosis by reducing ROS [24]. Here, we found that PPAR-*γ* activation induced the expression of Nrf2 in the primary rat chondrocyte pyroptosis primed with LPS/ATP, which counteracted inflammatory (Figures 2(f) and 3(h)) and oxidative damage (Figure 5(g)). Therefore, optimizing the activation of the PPAR-*γ*/Nrf2 pathway by screening pharmacological agonists seems possible to ameliorate OA cartilage destruction.

Mitochondria are targets of inflammatory and oxidative insults in the occurrence and development of OA [55]. It is known that the PPAR-*γ* and PGC-1*α* pathways could control inflammation and overcome mitochondrial impairment. PPAR-*γ* activated by Piog suppressed diabetes-induced atrial mitochondrial oxidative stress and protected mitochondrial biogenesis and *Δψ*_m_, which was blunted by PGC-1*α* siRNA transfection [29]. The PPAR-*γ*-dependent activation of PGC-1*α* signaling by glitazones is a plausible strategy to elevate antioxidative and anti-inflammatory responses as well as mitochondrial biogenesis for neuroprotection in various neurological conditions [27, 56]. Thiazolidinediones, including rosiglitazone and Piog, binding to GPR40 induced p38 MAPK phosphorylation and subsequently activated PGC-1*α* independent of PPAR-*γ* activation in the human endothelium. The reduction of the SIRT1/AMPK/PGC-1*α* and PPAR-*γ* signaling pathways by the perturbed homocysteine expression caused deleterious effects on chondrocytes, indicating a possible correlation between hyperhomocysteinemia and OA [57]. However, the crosstalk between these two signaling pathways was not elucidated. In this study, we found that PPAR-*γ* activation by Piog enhanced PGC-1*α* levels in chondrocytes primed with ATP/LPS. The PPAR-*γ* antagonist GW completely abolished an increase in the levels of PGC-1*α* upon Piog administration in the presence of ATP/LPS, which may be denied the involvement of the GPR40/p38 MAPK signaling pathway. In addition, the PGC-1*α* antagonist SR partially counteracted the effects of Piog on the NLRP3 inflammasome, ROS, and *Δψ*_m_. PGC-1*α* inhibited the activation of the NLRP3 inflammasome by protecting mitochondrial function, which was characterized in other diseases, such as kidney injury [58] and demyelinating disorders [59].

The increase in PGC-1*α* levels by the Nrf2 activator can regulate oligodendrocyte progenitor differentiation and mitochondrial biogenesis [21]. Contradictory scenarios were observed in certain cell types different from oligodendrocyte progenitors. PGC-1*α* may induce Nrf2 levels, and then inhibit the NLRP3 inflammasome to protect LPS/D-galactosamine-induced acute liver failure [60]. PGC-1*α* may also coactivate Nrf2 for the transcriptional activation of many antioxidant genes [61]. The downregulation of PGC-1*α* by metformin decreased Nrf2 levels by suppressing the transcriptional activity of PPAR-*γ* in MCF-7 breast cancer cells [62]. Nrf2 may negatively regulate PGC-1 transcriptional level. The PGC-1*α* mRNA level in the liver of Nrf2-knockout mice was significantly higher than that in the Nrf2-expressing mice [63]. Recent studies have reported the engagement of PGC-1*α* and NRF2 in cell pyroptosis, but the causal relationship between PGC-1*α* and NRF2 in cell pyroptosis was not scrutinized. Cadmium caused a reduced cerebral PGC-1*α* and NRF2 expression, increasing NLRP3 inflammasome-mediated pyroptosis in swine [64]. Chlorpyrifos activated pyroptosis and oxidative stress linked to nerve cell toxicity in SH-SY5Y cells by suppressing PGC-1*α* and Nrf2 level [65]. Downregulating miR-579-3p can protect the kidney against sepsis-induced injury by increasing PGC-1*α* and Nrf2 levels to reduce cell pyroptosis [66]. Here, we showed that Nrf2 and PGC-1*α* levels were induced by PPAR-*γ* activation upon Piog administration. Only concurrent inactivation of both Nrf2 and PGC-1*α* pathways could potently compromise the effect of Piog on NLRP3 inflammasome-mediated pyroptosis in rat chondrocytes. The additive effect probably exists between Nrf2 and PGC-1*α* pathways. Further investigation is required to determine whether the interplay between Nrf2 and PGC-1*α* partake in the suppression of chondrocyte pyroptosis of OA in response to PPAR-*γ* activation by Piog.

In conclusion, this study indicates that Piog protects the OA cartilage against pyroptosis-induced damage via PPAR-*γ* activation-dependent Nrf2 and the PGC-1*α* pathway, which enhances antioxidant and anti-inflammation response as well as mitochondrial biogenesis. The present results indicate that activation of both Nrf2 and PGC-1*α* by a combined modality of pharmacological agonists might serve as a potential option for OA treatment. Importantly, Piog, simultaneously activating Nrf2 and PGC-1*α*, is expected to be helpful for the therapeutic intervention in OA cartilage damage.

## Figures and Tables

**Table 1:** Histochemical assessment of rat articular cartilage changes.

Grade	S-O staining
0	Uniform staining throughout articular cartilage
1	Loss of staining in superficial zone of hyaline cartilage <50% the length of the condyle or plateau
2	Loss of staining in superficial zone of hyaline cartilage ≥50% the length of the condyle or plateau
3	Loss of staining in the upper 2/3's of hyaline cartilage <50% the length of the condyle or plateau
4	Loss of staining in the upper 2/3's hyaline cartilage ≥50% the length of the condyle or plateau
5	Loss of staining in all the hyaline cartilage <50% the length of the condyle or plateau
6	Loss of staining in all the hyaline cartilage ≥50% the length of the condyle or plateau

**Table 2:** OARSI score in each group.

Groups	OARSI
Control	0.33 ± 0.57
Piog	0.67 ± 0.58
OA	4.67 ± 1.15∗∗
OA + Piog	1.33 ± 1.15^##^

∗∗*P* < 0.01 versus control and ^##^*P* < 0.01 versus OA.

## Data Availability

The authors declare that all data supporting the findings of this study are available within the article.

## Supplementary Materials

Supplementary Materials The chemical structure of Piog is seen in Figure S1. The levels of Casp1-P10, GSDMD-N, and NLRP3 in all the OA samples are seen in Table S1.
