# Supplementary material for: PPAR-γ Activation Alleviates Osteoarthritis through Both the Nrf2/NLRP3 and PGC-1α/Δψm Pathways by Inhibiting Pyroptosis
Source: PPAR Res. 2023 Mar 27;2023:2523536. doi: 10.1155/2023/2523536 (PMC10070030; doi:10.1155/2023/2523536)
Supplement: Supplementary Materials — The chemical structure of Piog is seen in Figure S1. The levels of Casp1-P10, GSDMD-N, and NLRP3 in all the OA samples are seen in Table S1. [file 2523536.f1.docx]

**Supplementary materials**

**
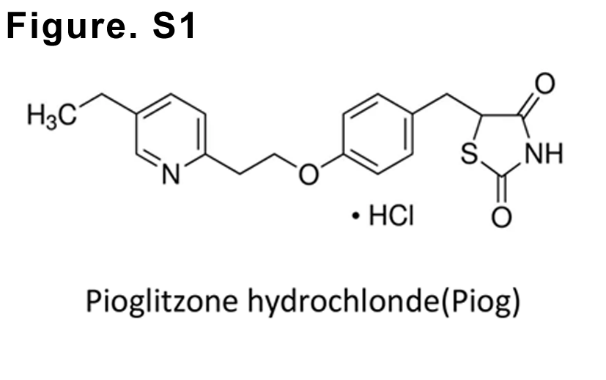
**

**Figure S1.** The chemical structure of Piog

**Table S1**. The levels of Casp1-P10, GSDMD-N, and NLRP3 in all the OA samples

|  | Mild (N=3) | Severe (N=3) | P |
| --- | --- | --- | --- |
| Casp1-P10 (IOD), mean ±SD | 3215.6±981.3 | 7397.9±1196.1 | 0.009 |
| GSDMD-N (IOD), mean ±SD | 2621.4±382.0 | 4645.2±291.7 | 0.002 |
| NLRP3(IOD), mean ±SD | 2440.8±744.6 | 4960.9±500.3 | 0.008 |
